# No Germs, Just Cytokines: A Case of Immunotherapy-Induced Hemophagocytic Lymphohistiocytosis Masquerading as Neutropenic Sepsis in a Patient With Inflammatory Breast Cancer

**DOI:** 10.7759/cureus.110906

**Published:** 2026-06-15

**Authors:** Nnaemeka H Ugwuchukwu

**Affiliations:** 1 Oncology, Antrim Area Hospital, Antrim, GBR

**Keywords:** hemophagocytic lymphohistiocytosis (hlh), immune checkpoint inhibitors (icis), immune-related adverse events (iraes), inflammatory breast cancer, neutropenic sepsis, pembrolizumab

## Abstract

Hemophagocytic lymphohistiocytosis (HLH) is a life-threatening hyperinflammatory condition leading to organ dysfunction and death. Primary HLH, driven by genetic mutations that impair immune regulation, predominantly affects children, whereas the secondary (acquired) form is more common in adults. Secondary HLH may be precipitated by various drivers, including medications such as immune checkpoint inhibitors (ICIs). Diagnosing this condition is difficult, as initial signs are often vague and overlap with those of other disorders; consequently, clinicians commonly use the Hemophagocytic Lymphohistiocytosis-2004 (HLH-2004) diagnostic criteria and the H-Score to support the diagnostic evaluation.

We report a case of a patient with triple-negative inflammatory breast cancer who developed HLH following anticancer therapy with pembrolizumab. The patient’s presentation with fever and cytopenias led to the initial management of neutropenic sepsis. However, following non-response to standard sepsis management, the patient was worked up for HLH. Diagnosis of HLH was supported by histopathological and laboratory findings; the patient fulfilled five HLH-2004 diagnostic criteria and responded to high-dose corticosteroid therapy.

This case highlights the importance of considering HLH as a rare immune-related adverse event (irAE) in patients receiving ICIs when presumed diagnosis fails to respond as expected. It further illustrates the value of applying validated diagnostic tools to expedite immunosuppressive therapy.

## Introduction

Hemophagocytic lymphohistiocytosis (HLH) is a rare, potentially life-threatening disorder, characterised by abnormal immune system activation, inflammation, and multi-organ failure [1,2]. While familial (primary) HLH (F-HLH) typically presents in childhood, HLH in adults is more often secondary to triggers such as infection, malignancy, autoimmune disease, or drug exposure, including immune checkpoint inhibitors (ICIs) [3,4].

The diagnosis of HLH remains challenging due to its nonspecific clinical presentation and the absence of a single definitive diagnostic test, necessitating reliance on complex criteria [5]. Patients commonly present with fever, organomegaly, neurological symptoms, and signs of liver dysfunction or coagulopathy [6]. These challenges contribute to a substantial rate of underdiagnosis leading to poor outcomes [5]. The Hemophagocytic Lymphohistiocytosis-2004 (HLH-2004) criteria established by the Histiocyte Society remains the most widely utilized and broadly accepted diagnostic tool for HLH [7].

ICIs, including anti-programmed cell death protein 1 (anti-PD-1) agents such as pembrolizumab, anti-programmed death-ligand 1 (anti-PD-L1) therapies, and anti-cytotoxic T-lymphocyte-associated antigen 4 (anti-CTLA-4) agents, have transformed cancer management by enhancing T-cell-mediated antitumor activity through the blockade of inhibitory immune pathways [4]. However, in recent years, reports of hyperinflammatory syndromes associated with ICI therapy have begun to emerge. Although these events are relatively uncommon, concerns that repeated exposure to a triggering agent may exacerbate HLH often prompt clinicians to discontinue treatment when this syndrome is suspected [8].

The association between ICIs and HLH is increasingly being recognised. This case illustrates the value of applying the HLH-2004 criteria early when neutropenic sepsis fails to respond to antibiotics. It also adds to the limited data on ICI-induced HLH in triple-negative breast cancer (TNBC).

## Case presentation

**Figure 1:**
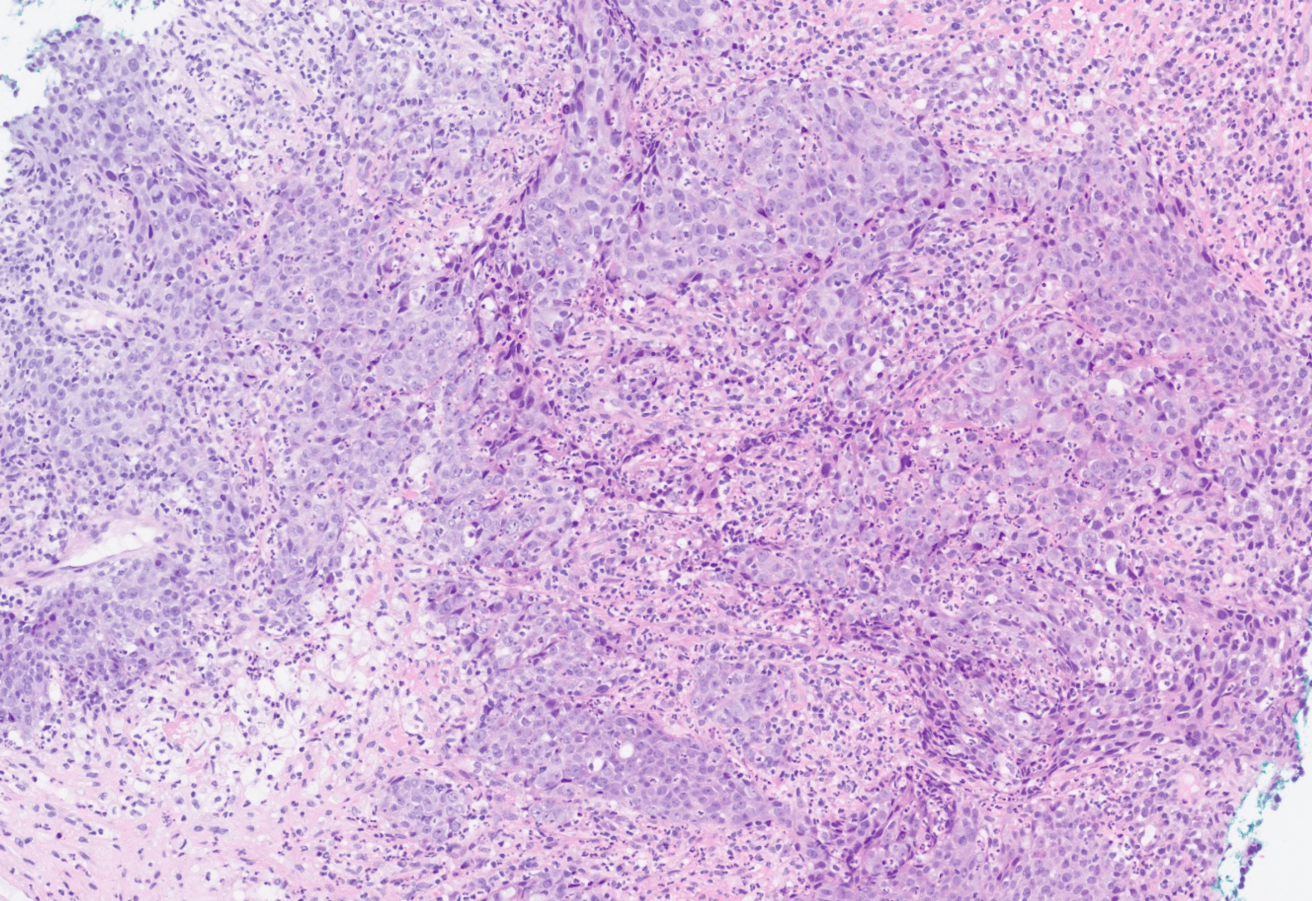
Histopathological Findings of Left Breast Core Needle Biopsy Specimen Representative hematoxylin and eosin (H&E) stained section at x10 magnification showing invasive carcinoma (no special type (NST)) with basal-like morphology. Receptor status (ER 5%, PR 0%, HER2 0%) was confirmed via separate immunohistochemical analysis (not shown).

Our patient was a 43-year-old woman diagnosed with inflammatory left breast cancer (57 mm), node-positive, grade 3 invasive carcinoma of no special type (NST), with low estrogen receptor expression (ER 5%) and negative progesterone (PR 0%) and human epidermal growth factor receptor 2 (HER2 0%) status, as shown in Figure 1. Following a multidisciplinary team meeting and discussion with the patient, the decision was made to treat as TNBC. She commenced neoadjuvant chemoimmunotherapy and, between May and August 2025, received pembrolizumab (five cycles) with carboplatin/paclitaxel (PC) (four cycles, May-July 2025), followed by epirubicin/cyclophosphamide (EC) (one cycle, August 2025).

She presented one week after her fifth cycle of pembrolizumab and first cycle of EC with a one-day history of fever. On examination, she was febrile (38.4) and tachycardic (pulse 113/minute). Abdominal examination demonstrated no splenomegaly, and the remainder of the systemic examination was unremarkable. Initial laboratory investigations showed bicytopenia with haemoglobin of 75 g/L and a white cell count of 0.5 × 10⁹/L (neutrophils 0.03 × 10⁹/L). She received one unit of packed red blood cells and was treated as neutropenic sepsis with intravenous (IV) broad-spectrum antibiotics. Although her fever initially improved, it recurred and persisted despite escalation of antimicrobial therapy. Computed tomography of the chest, abdomen, and pelvis showed no identifiable infectious focus or collections and demonstrated stable malignant disease without radiological evidence of progression. Blood cultures from both peripheral venous and peripherally inserted central catheter (PICC), PICC tip cultures, and urine culture for bacterial and fungal pathogens returned negative. Additionally, screenings for haematological malignancies and viral causes (including respiratory, human immunodeficiency, cytomegalovirus, Epstein-Barr, human T-lymphotropic, and hepatitis viruses) were negative, hence prompting our decision to investigate for HLH.

**Figure 2:**
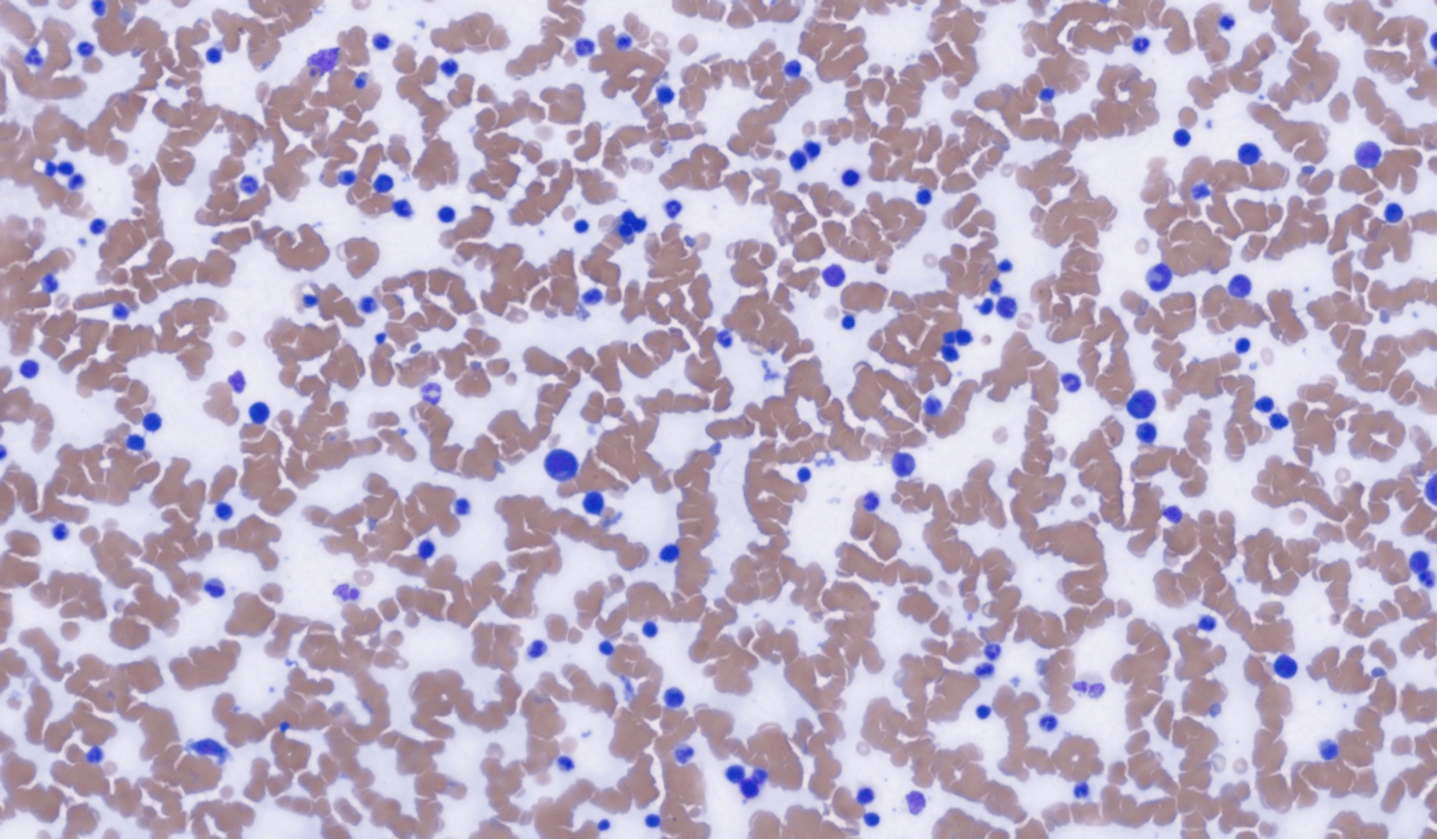
Bone Marrow Aspirate Findings Demonstrating Hemophagocytic Lymphohistiocytosis Representative bone marrow aspirate smear at x20 magnification showing a particulate, cellular sample with increased macrophage activity and frequent evidence of hemophagocytic lymphohistiocytosis (HLH). No abnormal lymphoid infiltration is seen.

Further evaluation for HLH revealed elevated inflammatory markers, including ferritin of 14,121 µg/L and triglycerides of 4.94 mmol/L. Bone marrow biopsy demonstrated increased macrophage activity with frequent hemophagocytosis, consistent with HLH. This is shown in Figure 2. Our patient fulfilled at least five HLH-2004 diagnostic criteria, including fever, cytopenia affecting two or more cell lines, hyperferritinaemia, hypertriglyceridemia, and evidence of hemophagocytosis. Additionally, calculated H-Score was 205, corresponding with an increased likelihood of hemophagocytic syndrome.

In the absence of an alternative precipitating factor and given the close temporal relationship to recent pembrolizumab exposure, HLH was attributed to ICI-associated immune dysregulation. She was commenced on IV methylprednisolone (1 g daily) for three days, followed by oral prednisolone at 60 mg daily for eight days. The oral dose was subsequently tapered by 10 mg every five days. Following corticosteroid initiation, the patient showed significant clinical improvement, marked by the resolution of fever and normalization of HLH-related laboratory parameters (Table 1). Pembrolizumab was permanently discontinued, and no rechallenge was undertaken.

**Table 1:** A summary of the evolution of haematological and biochemical parameters in relation to the initiation and tapering of steroids.

Parameter	Normal range	Admission	Day 0 IV methylprednisolone (admission day 20)	Day 1 IV methylprednisolone	Day 2 IV methylprednisolone	Day 0 oral prednisolone (admission day 23)	Discharge (admission day 30; weaning steroids)	Follow-up (2 weeks)
Haemoglobin (g/L)	120-150	75	106	99	93	103	107	124
WCC (×10⁹/L)	4-10	0.5	8.9	3.2	3.7	6.1	8	16.1
Neutrophils (×10⁹/L)	2.0-7.0	0.03	6.25	1.9	2.33	4.72	6.42	15.39
Platelets (×10⁹/L)	150-450	184	93	98	141	226	260	230
Triglycerides (mmol/L)	1.0-2.50	-	4.94	4.67	3.74	6.57	7.87	2.73
Lactate Dehydrogenase, LDH (U/L)	135-214	240	-	-	-	-	-	-
Aspartate Aminotransferase, AST (U/L)	0-32	20	33	29	23	-	-	-
Alanine Aminotransferase, ALT (U/L)	0-33	33	47	44	39	-	63	-
Alkaline Phosphatase, ALP (U/L)	30-130	98	95	97	95	115	130	213
Ferritin (µg/L)	15-150	-	14,121	8,616	5,560	2,722	1,364	560
Fibrinogen (g/L)	1.8-4.3	-	4.47	4.47	3.06	2.05	1.73	3.42

## Discussion

Secondary HLH is a hyperinflammatory emergency characterised by uncontrolled immune activation and cytokine excess, culminating in progressive organ dysfunction and high mortality if not recognised early [1,9]. While most reported cases are associated with malignancies (notably melanoma and lung cancer), infections, or autoimmune disorders, the increasing use of ICIs such as pembrolizumab in breast cancer has been linked to immune-related adverse events (irAEs), including HLH in this patient population [4,10,11].

Adult HLH is a rare and under-characterised condition with limited epidemiological data [6]. It typically affects middle-aged adults, and has a general male predominance, except for macrophage activation syndrome (MAS), which is more commonly seen in women [12,13]. HLH can occur from the first immunotherapy cycle to weeks after cessation, requiring continued monitoring. With pembrolizumab, the reported median onset is 12 weeks (range 1-74 weeks) [14]. Increasing recognition of secondary HLH is reflected in emerging case reports, further highlighting its evolving clinical spectrum and growing association with modern immunotherapies [4]. Notably, published cases in breast cancer have been reported since 2018 [12].

The exact pathophysiological mechanisms underlying secondary HLH are not fully defined and are thought to involve a range of contributing factors [3]. Some studies have reported that nearly 15% of cases may carry heterozygous variants in genes commonly associated with F-HLH, which do not result in complete loss of protein function [3,15]. However, Carvelli et al. found no evidence of impaired cytotoxic function in patients with secondary HLH carrying monoallelic mutations in genes associated with F-HLH, thereby creating uncertainty whether these mutations have any role in the pathogenesis of acquired HLH [16].

Secondary HLH is driven by excessive proinflammatory cytokine production, and in the context of immune checkpoint inhibition, is thought to arise from dysregulated hyperactivation of cytotoxic CD8+ T cells, culminating in a cytokine storm and tissue injury [3,14]. Under normal physiological conditions, PD-1 signalling acts as a key regulatory brake on T-cell activity, maintaining self-tolerance and limiting autoimmunity. However, tumour cells may exploit this pathway via upregulation of PD-L1, thereby attenuating effective antitumor immune responses. Pembrolizumab, through its disruption of PD-1/PD-L1 signalling, restores cytotoxic T-cell activity and augments immune-mediated tumour eradication [4,14].

Early recognition of secondary HLH is essential, given that patients are often very unwell and diagnostic delays are associated with adverse outcomes; nevertheless, diagnosis remains difficult because its clinical features frequently overlap with conditions such as sepsis, chemotherapy-related toxicities, and progression of malignant disease. While chemotherapy-induced bone marrow suppression and neutropenic sepsis are important initial differential diagnoses in our patient, given the recent administration of EC, the absence of an identifiable infectious source despite extensive investigations, together with persistent fever and a lack of clinical improvement with antimicrobial therapy and neutrophil recovery, prompted consideration of HLH.

The HLH-2004 criteria and the H-Score are two widely recognised diagnostic tools used in the evaluation of HLH [3,4]. The HLH-2004 framework establishes a diagnosis when at least five of eight criteria are met [6], whereas the H-Score estimates the likelihood of HLH using nine parameters, with a diagnostic threshold of 169 [3]. In this case, our patient fulfilled five of the eight HLH-2004 criteria (fever, cytopenias, hyperferritinemia, hypertriglyceridemia, hemophagocytosis) and recorded an H-Score of 205, both of which strongly support the diagnosis of HLH.

The primary aim of management of ICI-induced HLH is to halt the excessive and damaging hyperinflammatory immune response. In most cases, the first line of treatment typically involves stopping the suspected triggering agent and initiating corticosteroid therapy [17]. Several reports have demonstrated favourable outcomes with corticosteroid monotherapy alone. However, in refractory or severe cases, additional immunosuppressive agents such as cyclophosphamide or etoposide may be required [4,18]. More recently, interleukin-6 (IL-6) receptor antagonists such as tocilizumab have shown promising therapeutic potential in ICI-induced HLH, with some studies proposing their concurrent use with ICIs to mitigate irAEs while preserving antitumor efficacy [18].

## Conclusions

ICI-associated HLH is a rare but potentially life-threatening irAE that should be considered in patients presenting with persistent fever, cytopenias and signs of systemic inflammation following immunotherapy. Early use of established criteria can support timely diagnosis and initiation of immunosuppression.

This case adds to the growing reports of HLH in breast cancer patients receiving ICIs. It highlights the need for further real-world data to inform optimal treatment strategies and decisions regarding ICI interruption or rechallenge. Whether ICIs can be safely rechallenged after HLH resolution remains uncertain and requires individual risk-benefit assessment.
